# First Evidence of Chelonid Herpesvirus 5 (ChHV5) Infection in Green Turtles (*Chelonia mydas*) from Sabah, Borneo

**DOI:** 10.3390/pathogens10111404

**Published:** 2021-10-29

**Authors:** Aswini Leela Loganathan, Pushpa Palaniappan, Vijay Kumar Subbiah

**Affiliations:** 1 Biotechnology Research Institute, Universiti Malaysia Sabah, Kota Kinabalu 88400, Sabah, Malaysia; aswinileela7@gmail.com; 2 Genomics Facility, Monash University Malaysia, Bandar Sunway 47500, Selangor, Malaysia; 3 Borneo Marine Research Institute, Universiti Malaysia Sabah, Kota Kinabalu 88400, Sabah, Malaysia; pushpa@ums.edu.my

**Keywords:** Borneo, ChHV5, *Chelonia mydas*, fibropapillomatosis, Mabul Island, Sabah, Malaysia

## Abstract

Fibropapillomatosis (FP) of sea turtles is characterised by cutaneous tumours and is associated with Chelonid herpesvirus 5 (ChHV5), an alphaherpesvirus from the family Herpesviridae. Here, we provide the first evidence of ChHV5-associated FP in endangered Green turtles (*Chelonia mydas*) from Sabah, which is located at the northern region of Malaysian Borneo. The aims of our study were firstly, to determine the presence of ChHV5 in both tumour exhibiting and tumour-free turtles using molecular techniques and secondly, to determine the phylogeography of ChHV5 in Sabah. We also aim to provide evidence of ChHV5 infection through histopathological examinations. A total of 115 Green turtles were sampled from Mabul Island, Sabah. We observed three Green turtles that exhibited FP tumours and were positive for ChHV5. In addition, six clinically healthy turtles (with no presence of tumours) were also positive for the virus based on Polymerase Chain Reaction of three viral genes (Capsid protein gene UL18, Glycoprotein H gene UL22, and Glycoprotein B gene UL27). The prevalence of the ChHV5 was 5.22% in asymptomatic Green turtles. Epidermal intranuclear inclusions were identified in tumour lesions upon histopathological examination. In addition, phylogenetic analyses of the UL18, UL22, UL27, and UL30 gene sequences showed a worldwide distribution of the ChHV5 strain with no clear distinction based on geographical location suggesting an interoceanic connection and movement of the sea turtles. Thus, the emergence of ChHV5 in Green turtles in the waters of Sabah could indicate a possible threat to sea turtle populations in the future and requires further monitoring of the populations along the Bornean coast.

## 1. Introduction

Fibropapillomatosis (FP) is a debilitating neoplastic disease of sea turtles [1,2]. The first reported case of FP was in Florida in 1938 where a Green turtle was captured with tumour-like characteristics [3]. However, it has been reported that disease outbreaks in the wild have increased since the 1980s [4]. In Asia, it was reported that FP tumour was first observed in nesting Green turtles (*Chelonia mydas*) from Sarawak Turtle Islands in 1958, but there have been no further reports of possible etiological agents in the waters of Malaysia [5]. However, a high prevalence of FP was observed in Indonesia, a neighbouring country to Malaysia [6]. In addition, FP has been classified as a pandemic disease which appears to be increasing in some parts of the world [7]. This further serves a need to determine the presence of FP and to better understand how it might affect turtle populations in Borneo, a region where the health of sea turtles is poorly understood.

FP of sea turtles is described as a neoplastic condition where it is clinically characterised by tumour formation in both external (skin) and internally (organs) [7,8]. The tumour primarily occurs on the eyes, soft tissues, flippers, carapace, plastron, and internal organs and the size may reach 20 cm in diameter [5,9]. The affected sea turtles become thin and weak and their daily activities such as feeding and locomotion are hampered as the tumour begins to impede organ functions. Eventually the tumours grow large enough and impair buoyancy of the turtle and in several cases cause death [4,7,9,10].

As severe tumour loads could be life threatening to the sea turtles, FP has emerged as an important panzootic disease that needs to be monitored [10,11]. While the cofactor of the outbreaks from Hawaii, Florida, and Indonesia has been hypothesised to be environmental factors, metazoan parasites, and oncogenic viruses, the etiologic agent most consistently associated with FP is Chelonid herpesvirus 5 (ChHV5), an alphaherpesvirus from the family Herpesviridae [5,8,10,12,13,14]. As such, a thorough understanding of the cause and pathogenesis is needed to aid in controlling efforts against the disease [10].

ChHV5 infections, as with most other herpesvirus infections, result in latent infections that persist over time. Even though the virus is dormant, the virus transmission is reactivated under certain circumstances such as temperature, environmental change, and efficacy of the host’s immune system [5,10,11,14]. Consequently, infected sea turtles may be carrying segments of the viral DNA without exhibiting any signs of tumours in the initial stages of infection [4,15]. This implies that environmental factors and other putative causes enhance the virus outbreak and facilitate transmission. At times, tumours develop large enough to cause death [4,16].

The complete genome of ChHV5 for an infected turtle was sequenced using BAC-derived sequences and is known to have a type D genome [17]. Several studies have used classic molecular approaches to detect ChHV5 DNA via polymerase chain reaction (PCR) in tumour samples from turtles affected with FP [9,10,14]. Replication of the herpesviruses is common in tissues that exhibit tumours as large concentrations of viral particles exist. Hence, the expression of ChHV5 in these turtles is predictable. However, during latent infections, the concentrations of viral particles are very low, presenting a challenge to detect the virus [4,14].

Although FP has been documented worldwide, reports of this condition for sea turtles in Asiatic waters are limited [5,18]. There is anecdotal evidence of Green turtles and Hawksbill turtles (*Eretmochelys imbricata*) with FP tumours in northeastern Borneo but the occurrence of ChHV5 have not been identified in these ecologically rich waters. The purpose of our study was to determine the presence of ChHV5 in both tumour exhibiting and tumour-free turtles using molecular techniques and to determine the phylogeography of ChHV5 in the Sabah region. To our knowledge, we provide the first records of ChHV5 in Green turtles from Borneo.

## 2. Results and Discussion

**Figure 1:**
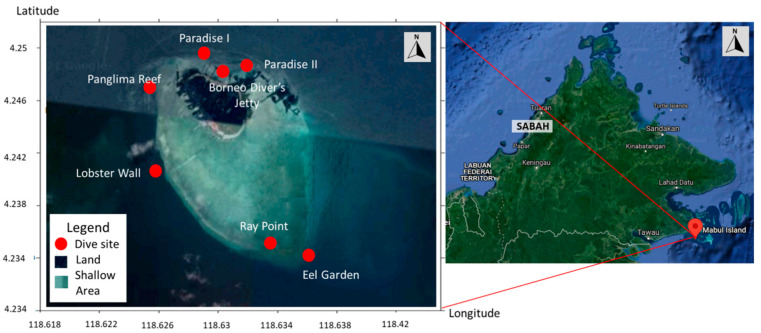
Map of Mabul Island indicating the dive sites where sea turtles were captured. The inset (**left**) shows the location of the island at northern Borneo at the south-eastern region of Sabah, Malaysia. Mabul island (**right**) is highlighted in dark green while the surrounding shallow area in light green (image taken from Google map).

We sampled a total of 131 living turtles (115 Green and 16 Hawksbill turtles) in the waters of Mabul Island during four-day trips in May and November 2015 and November 2016 (Figure 1). The island, along with its well-known neighbour Sipadan Island, is a foraging ground for Green and Hawksbill turtles. Most of the turtles were deemed clinically healthy (i.e., no signs of FP tumours) with overall good body condition (convex-shaped plastrons) and size. Green turtles were mainly juveniles with curved carapace lengths ranging from 387 to 1075 mm and more abundant than Hawksbills (1 Hawksbill:7 Green turtle ratio). Meanwhile, the Hawksbill turtles had curved carapace lengths ranging from 410 to 824 mm.

Three Green turtles with visible FP tumours were encountered at Mabul Island. The tumour scores were defined based on the study proposed by Work and Balazs [19] where the tumours were classified into four categories. Size A (<1 cm), Size B (1–4 cm), Size C (4–10 cm), and finally Size D (>10 cm). In our study, we found tumours comprising all four categories. The largest tumour observed was 12 cm in size which was present on the ventral surface of the turtle. Variation in size and morphology of the tumours may depend on geographical regions and where some parts of the world may have a higher percentage of corneal FP while other parts may only have epidermal tumours [20].

The relative low prevalence of FP tumours (2.6%) at Mabul Island among the Green turtles is in stark contrast to reports from other localities. For example, in neighbouring Indonesia the overall prevalence of FP was 21.5% [6]. Meanwhile, other areas such as the Indian River Lagoon (FL, USA), Kaneohe Bay (HI, USA), and Moreton Bay (Australia) have reported very high prevalence of more than 50% [20]. It is highly likely that the low prevalence at Mabul Island is due to the pristine crystal clear conditions of the waters with little pollution and human activities. This is in contrast with the report from Indonesia where FP turtles were found mostly in densely populated and industrial regions [6]. It would seem to suggest that low water quality and adverse environmental factors may be a triggering factor here.

**Figure 2:**
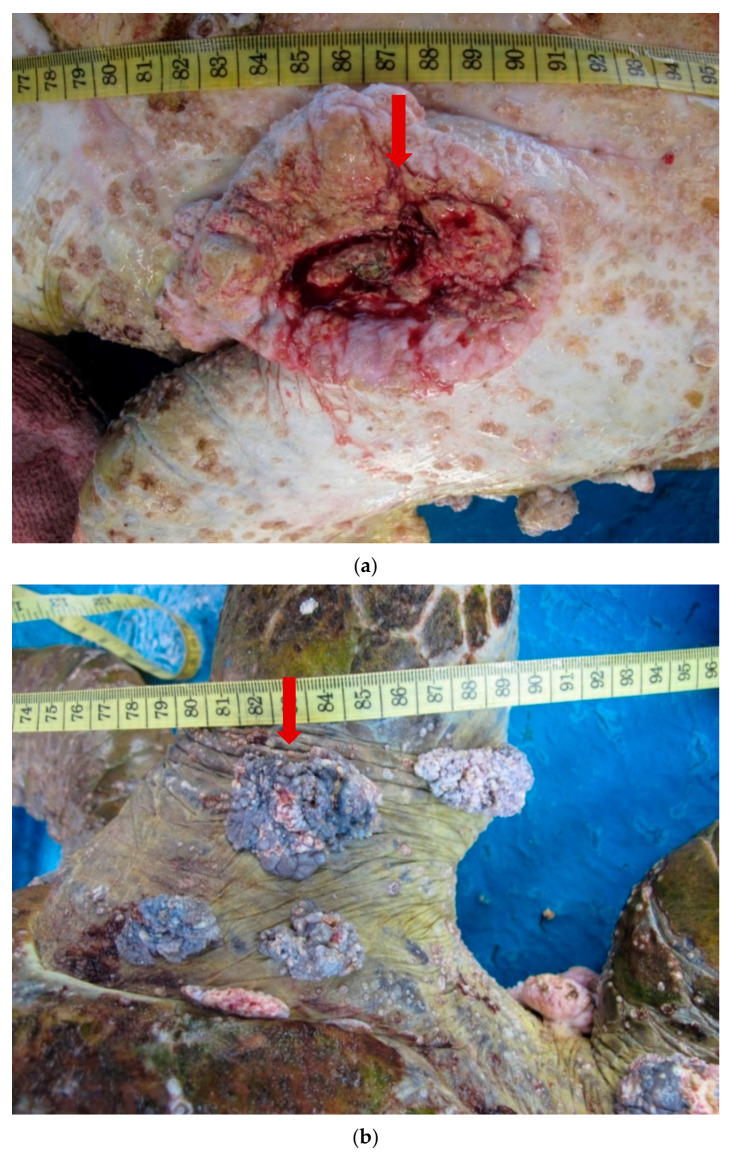

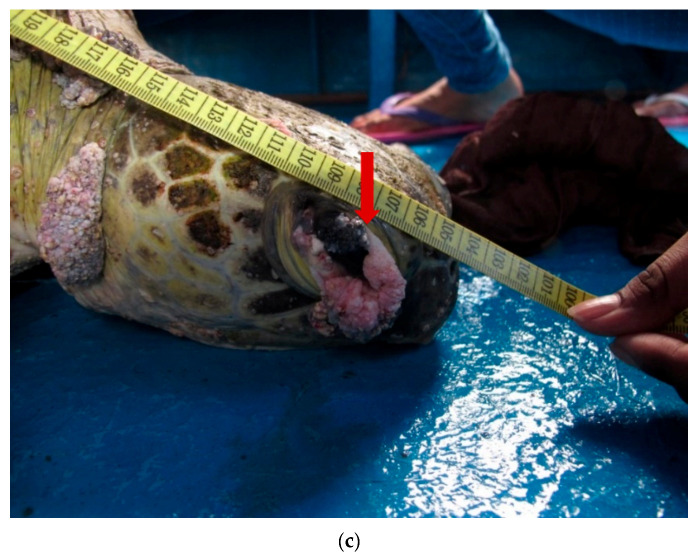
(**a**). A 12 cm tumour (arrow) present on the ventral surface of a Green turtle. (**b**). Multiple verrucous nodules (arrow) on the dorsal surface of the hind flipper of a Green turtle. (**c**). A 4 cm verrucous lesion at the lateral canthus of the eye of a Green turtle.

We observed that morphologically, all the tumours were a combination of either smooth or verrucous. Most tumours were soft while some appeared to be rigid. The nodules were firm and pale, with similar morphology to those described in earlier studies [8,19]. One individual had a debilitating advanced stage of FP with tumours in all regions of the body (hind flippers, plastron, neck, and carapace) (Figure 2a,b). Multiple nodules, ranging from 1 to 7 cm in diameter, were present on the ventral surfaces of both back flippers. The nodules contained numerous leeches, but these parasites were not examined in the current study. Two of the turtles had tumours on the eye lid (Figure 2c) which impacted their vision.

**Figure 3:**
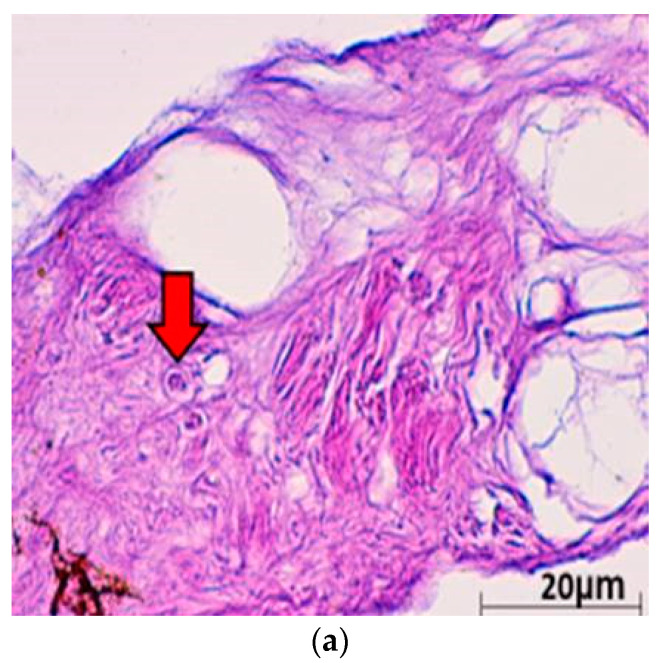

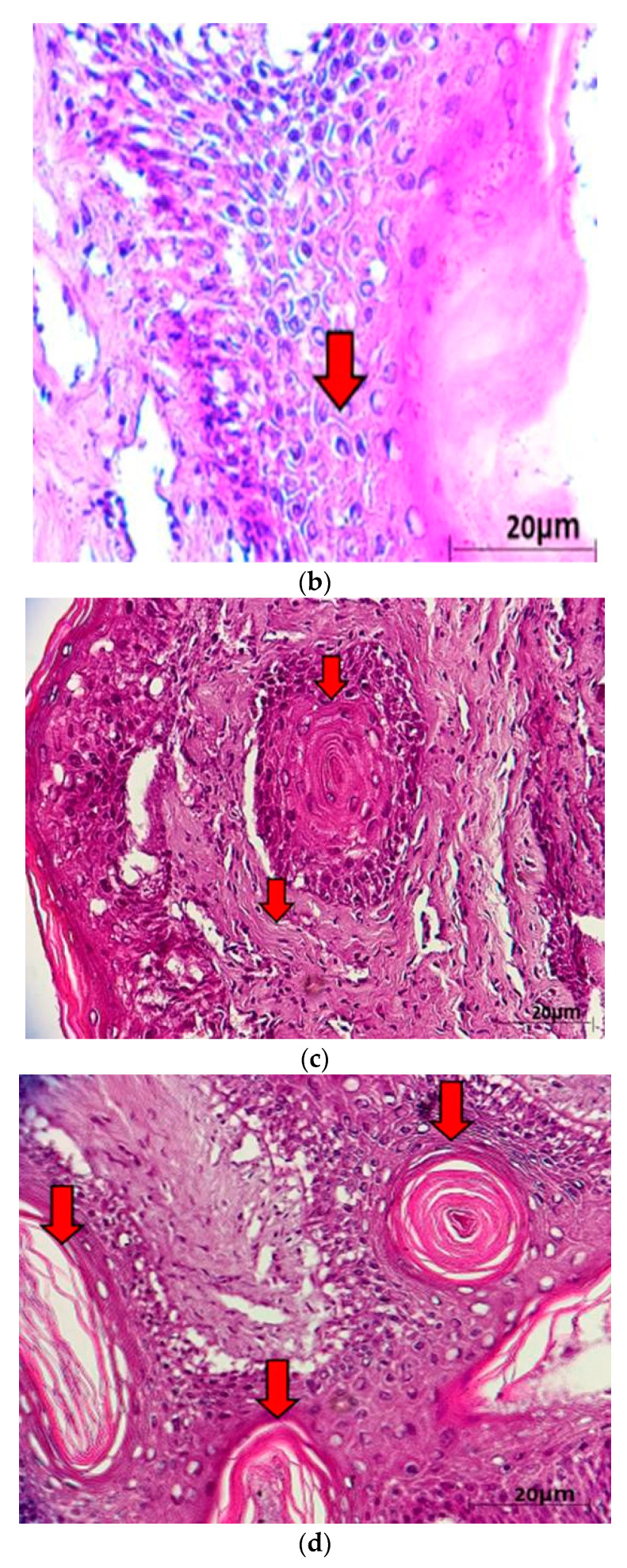
(**a**) Histological cross section of one of the verrucous nodulations sampled from the ventral surface of the hind flipper of a Green turtle where it shows epidermal internuclear inclusions (arrow) within epidermis manifesting ballooning degenerations (40× magnification with haematoxylin and eosin stain; scale bar = 20 µm). (**b**) Histological cross section of one of the verrucous nodulations sampled from a Green turtle where it shows Acanthosis and orthokeratotic hyperkeratosis with necrosis of the stratum basale (arrow) (40× magnification with haematoxylin and eosin stain; scale bar = 20 µm). (**c**) Histological cross section of one of the verrucous nodulations sampled from the ventral surface of the hind flipper of a Green turtle where it shows epidermal hyperplasia (arrow) (40× magnification with haematoxylin and eosin stain; scale bar = 20 µm). (**d**) Histological cross section of one of the verrucous nodulations sampled from the ventral surface of a Green turtle where it shows Horn Cyst (arrow) (40× magnification with haematoxylin and eosin stain; scale bar = 20 µm).

Histopathological examinations were conducted to provide evidence of ChHV5 in tumour tissue. Light microscopic examination of the tumour lesion that was biopsied revealed the presence of Horn Cyst (Figure 3). Hyperkeratotic epidermis (characterised as papilloma) and acanthosis with Epidermal intracellular inclusion bodies (EIIs) were also identified (Figure 3a–c). Horn Cysts indicate a viral infection and confirm the presence of ChHV5 in the tumours [21]. The Hyperkeratotic epidermis and acanthosis with epidermal intracellular inclusion bodies (EIIs) implies a transcriptionally active (i.e., non-latent) state of ChHV5 in FP tumours of the affected Green turtles. IILs are commonly found on the outer layer of the epidermis [22]. Therefore, it is transmitted easily when in contact with other foraging turtles or spread into the environment. This observation is consistent with the previous reports for FP in sea turtles [22,23,24], and further supports the initial hypothesis by Jacobson et al. [25] that the virus is replicating in FP tumours from sea turtles. The results seem to indicate that ChHV5 plays an active role in FP of Green turtles and could be horizontally transmitted between regions based on the phylogenetic analysis.

In addition to the gross and histopathological examinations, molecular analysis was conducted to provide additional evidence of ChHV5 infection. A total of 131 (115 Green and 16 Hawksbill) individual sea turtles were screened for ChHV5 (including the three with visible signs of tumour) using PCR by amplifying sequences that represent the four viral genes (Capsid protein gene UL18, Glycoprotein H gene UL22, Glycoprotein B gene UL27, and DNA polymerase catalytic subunit pol UL30). All 16 Hawksbill turtles tested negative for the ChHV5 virus. Of the 115 Green turtles analysed (FP-exhibiting and clinically healthy), nine Green turtles (3 FP exhibiting and 6 clinically healthy turtles) were PCR-positive for ChHV5 (Table 1). Four samples were positive for the Capsid protein gene UL18, nine samples were positive for the Glycoprotein H UL22 gene, while six samples had sequences of the Glycoprotein B gene UL27. As for the DNA Polymerase gene UL30, five positive samples were detected (Table 1). A previous study by Alfaro-Nunez and Gilbert [15] concluded that, even though the sea turtles are infected with ChHV5 virus, it is very unlikely to indicate PCR-positive if only a single gene is used to screen for the presence of the virus. Consequently, the combination of all the four genes allowed the screening of ChHV5 positive samples with differing stages of infection.

**Figure 4:**
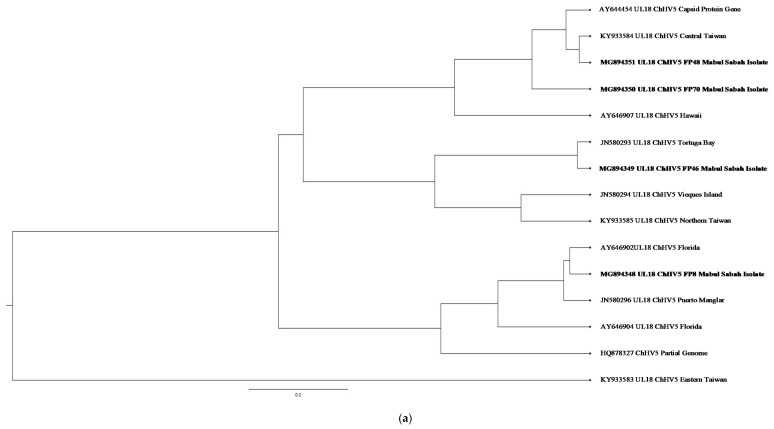

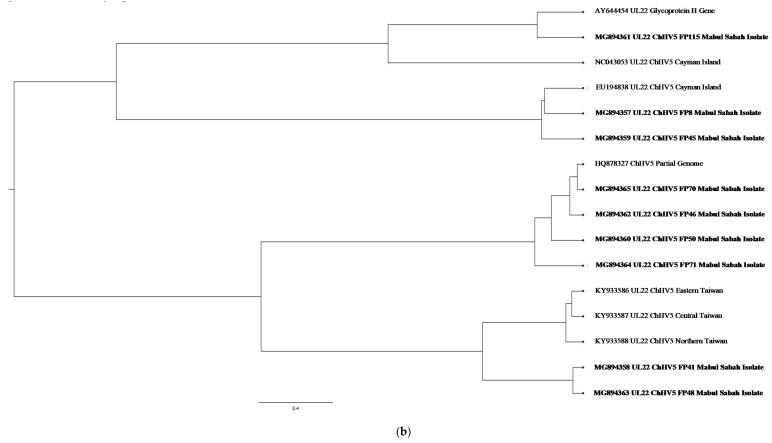

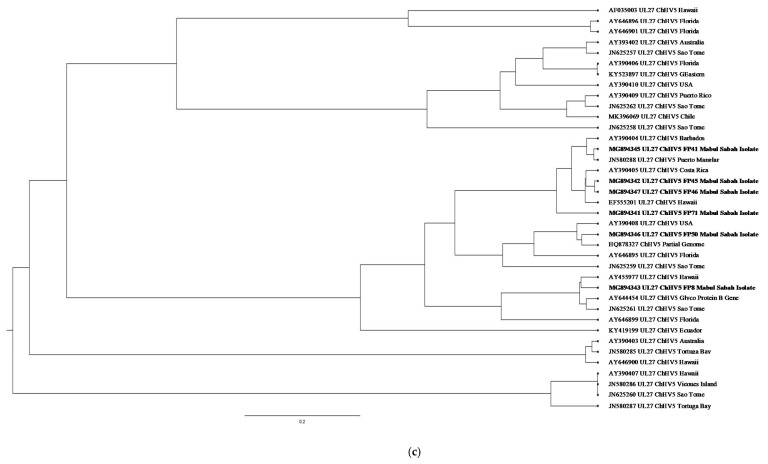

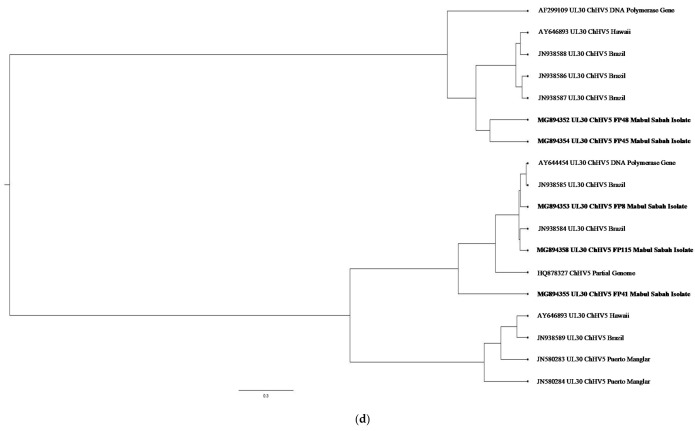
(**a**) Cluster analysis and distribution of ChHV5 variants inferred using BEAST v2.50. The ChHV5 Major capsid protein gene (UL18) from Mabul Island indicates that the Mabul Island strain is clustered closely to the Brazilian strain of Vieques Island, Puerto Manglar, and Tortuga Bay. (**b**) Cluster analysis and distribution of ChHV5 variants inferred using BEAST v2.50. The ChHV5 glycoprotein H gene sequences (UL22) indicate that the Mabul Island strain is clustered together with those from Cayman Island. Four viral sequence of the ChHV5 glycoprotein H gene sequences (UL22) from Mabul Island strain were clustered into a separate branch. (**c**) Cluster analysis and distribution of ChHV5 variants inferred using BEAST v2.50. The Glycoprotein B (UL27) gene sequences indicate that the Mabul Island strain has more similarity to the Hawaiian and Brazilian strains. (**d**) Cluster analysis and distribution of ChHV5 variants inferred using BEAST v2.50. Phylogenetic tree of UL30 DNA polymerase gene shows that the ChHV5 variants from Mabul Island are closely related to the variants from Florida.

Various hypotheses have been suggested for ChHV5 to be present in both FP-exhibiting and clinically healthy turtles. At present, latent infection is the most accepted hypothesis for these conditions. Like most Herpesvirus infection characteristics, the virus is capable of causing latent infections in their host [20,26]. This characteristic plays a crucial role during latent infections, as the viral gene is still present but the expression is minimised. By using four genic regions for the detection of ChHV5, Alfaro-Núñez and Gilbert [15] hypothesised that the detection accuracy is proportional to the viral DNA concentration and the sizes of the tumour sample. Similarly, the ability of the viral DNA to anneal to the primers during PCR plays a major factor as well. As such, it presents a challenge in detecting ChHV5 in latent infections. Hence, an absence of tumour does not necessarily indicate the absence of ChHV5 infection. This hypothesis was supported by previous research conducted by Alfaro-Nunez et al. [1] and Jones et al. [11]. All ChHV5 positive sequences from our study have been deposited in GenBank (https://www.ncbi.nlm.nih.gov, accessed on 7 December 2020) with the accession numbers MG894341-MG894365. Phylogenetic analysis inferred using the ML method showed clusters and distribution of the ChHV5 strain from Mabul Island and isolates from other geographical regions. The phylogenetic tree was constructed from the sequences obtained from BLAST nucleotide search. The phylogenetic trees were constructed with capsid protein gene (UL18), glycoprotein H gene (UL22), glycoprotein B (UL27), and DNA polymerase gene (UL30) sequences (Figure 4a–d).

Phylogenetic analysis inferred using BEAST v2,50 and visualised using FigTree software (http://tree.bio.ed.ac.uk/software/figtree/, accessed on 19 May 2020) showed clusters and distribution of the ChHV5 strain from Mabul Island and isolates from other geographical regions. The ChHV5 Major capsid protein gene (UL18) from Mabul Island indicates that the Mabul Island strain is clustered closely to the Brazilian strain of Vieques Island, Puerto Manglar, and Tortuga Bay.

On the other hand, the ChHV5 glycoprotein H gene sequences (UL22) indicate that the Mabul Island strain is clustered together with those from Cayman Island. Four viral sequences of the ChHV5 glycoprotein H gene sequences (UL22) from Mabul Island strain were clustered into a separate branch. Some of the viral sequence of ChHV5 major capsid protein gene (UL18) and ChHV5 glycoprotein h gene (UL22) from Mabul island followed similar pattern, clustering with the viral strain from Taiwan. A recent study by Li et al. [5] compared the Glycoprotein B gene from Taiwan, indicating that the Taiwanese strain was also grouped with those from Hawaii, Sao Tome, and Puerto Rico, which is similar to the Mabul strain.

The Glycoprotein B (UL27) gene sequences have more similarity to the Hawaiian and Brazilian strains. This could be due to interoceanic connection and admixture of populations. Although, further analysis is needed to support this hypothesis. As for the DNA polymerase gene (UL30), the phylogenetic analysis showed that most of the Mabul Island strain is clustered together with the Brazilian strain.

The general result inferred from the phylogenetic tree may not represent the full genetic distribution based on geographical location as the study is based on a small sample size. However, the phylogenetic tree generally infers the movement of the sea turtle host reflecting if the ChHV5 have undergone region-specific co-evolution through the sea turtle host. The phylogenetic analysis is also important to identify the differences in gross manifestation across different geographical regions. For example, there is a higher prevalence of oral tumours in Hawaii compared to Florida which has zero cases of oral tumour [27].

As for the Mabul strains, although the ChHV5 viral strain were clustered together with the Hawaii strain, there were no oral tumours found morphologically. Nevertheless, the ability for the virus to cause latent infection, where there is minimum expression of the viral gene, should be considered an important aspect. Furthermore, the tumour formation could be induced in specific tissue and absent in other tissues [1]. As the phylogenetic result showed that there is no clear distinction based on geographical location, the interaction between host, pathogen, and environment should be considered.

A previous study by Greenblatt et al. [20] implied that sea turtles may have a passive infection where the pathogenesis of the infection depends on environmental triggers such as pollutants and marine leeches. Similarly, Tristan et al. [28] reported that sea turtles living in habitats that are nearby urban development and are of close proximity with human contact have increasing chances to develop the tumours.

A possible explanation from a previous study is that the sea turtles may have come in contact with environmental pathogens such as parasitic leeches during the migration period (pelagic phase) of the sea turtles, where the turtles are capable of migrating hundreds to thousands of kilometres to different geographical regions in search of foraging grounds. Although previous studies have reported that sea turtles are infected with ChHV5 after returning to neritic habitat from pelagic stage, Rittenburg et al. [29] suggested that the development of FP is influenced by complex interconnected factors such as presence of vectors, super spreaders, and transmission through the environment. Hence, the host response and transmission of the virus varies across populations [29,30,31].

A study by Work et al. [31] suggested that the epizootiology of FP is associated with the life cycle of sea turtles. The hatchlings spend years in the pelagic (open ocean) environment and find a neritic (nearshore) foraging ground as juveniles and sub-adults [30,31,32]. The juveniles acquire ChHV5 infection soon after migrating to foraging ground from pelagic environment through direct contact or leeches [31,32]. Whether the hatchlings in the pelagic phase are infected with FP before migrating to the neritic environment is still understudied.

Ene et al. [32] reported that the occurrence of tumours in juvenile turtles at neritic habitat could be from prolonged tumour development due to virus infection or environmental triggers. Therefore, infection of ChHV5 could occur either through maternal shedding from the natal beach, pelagic phase, or neritic phase. If the hatchlings are infected from the natal beach, then there are high possibilities for them to carry latent infection into the pelagic phase [32]. Hence, there are different views and opinions regarding the attribution of the virus. Furthermore, as the precise role of ChHV5 has not been elucidated the transmission of viral particles during the pelagic stage remains debatable. These parasitic leeches have been suggested to be the mechanical vector for ChHV5 in Green turtles [33]. It was also documented that the viral particle could survive in the ocean for a short period of time before the virus capsid protein degrades [1]. Environment co-factors such as water temperature and habitat degradation have been suggested to play a role in FP pathogenesis [34,35]. Similarly, viral latency, viral load thresholds, and other ecological factors contribute in the tumour expression [30,35].

Although we observed parasitic leeches in the tumours of the turtles, unfortunately we did not perform further examination, which may serve as a limitation of our study. Other co-factors such as environmental (in addition to the parasitic leeches) could contribute and influence the expression of virus. We suggest that future study should include a larger sample size together with the analysis of the parasitic leech to further support this hypothesis.

Herpesviruses are horizontally transmitted from one infected host to another by bodily fluid such as saliva, mucus, or direct physical contact [15,22]. Horizontal transmission of ChHV5 has been reported to occur through viral particle shredding in the water [36,37]. The dispersal and virulence of the viral particle are influenced by the water temperature. It was reported that herpesvirus remains infectious at 23–30 °C as higher temperatures are documented to enhance herpesviruses replication [36,37].

Hence, the virus from the infected turtle may be transmitted to healthy sea turtles. This is made possible as the juvenile and sub-adult sea turtles undergo long distance migrations known as pelagic life stage, migrating between the juvenile nursery habitat and developmental areas while residing there for years [38]. Furthermore, as sea turtles are highly migratory animals, their habitual place differs distinctly through different life stages and FP prevalence varies among species and regions [30]. Thus, there is a high probability that the sea turtles acquired the infection during this period of their life cycle. Hence, in a foraging ground, the population of sea turtles may be from different geographical regions. This may be the reason why only a certain number of the sea turtles in a population develop ChHV5 [28] and may explain why the Mabul island ChHV5 viral strain are generally clustered together with different populations from different regions such as the Taiwan, Brazilian, and Hawaiian viral strain, respectively.

Jones et al. [11] suggested that the variability in host immunity and geographic regions could influence the viral variants of an infection. To date there are six variants documented for Chelonid herpesvirus (ChHV5). ChHV5 1, 5, and 6 are designated for marine turtles and ChHV5 2, 3, and 4 are described for freshwater turtles. The variants of a virus could have diverse virulence levels and as such the severity and disease presentation may differ with each variant and could become geographically specific.

Furthermore, the movement of the virus (sea turtle host) to these regions could be clarified by the strong equatorial ocean current that supports the flow of the virus as suggested by Patricio et al. [4]. In support of this, Putman and Naro-Maciel [39] stated that the particles in the ocean follow the movement of TransAtlantic dispersion of sea turtles that involves persistent connectivity between the southwestern Indian Ocean and the South Atlantic. This shows that the oceanic currents play a crucial role as they carry the turtle hatchlings through different geographical regions where several of these turtles end up foraging in the region until they mature. Sea turtles from the Bornean waters are known to follow the oceanic drift by the Celebes and Sulu Seas [40,41]. Although the hatchlings and young juveniles follow the ocean current to drift between the foraging areas, some adult turtles have been known to swim against the current, hence resulting in an undefined population group. This is because sea turtles are also known to be influenced by ontogenetic habitat shifts [42,43]. Snover [42] stated an example of post-larvae which may settle from the pelagic environment to benthic habitats that serve as early juvenile habitats and eventually to adult habitats where they move from pelagic to neritic habitats.

Additionally, previous studies have suggested that the cold waters at the tip of Africa are a barrier for the dispersal of sea turtles from the Atlantic Ocean to the Indian Ocean [44,45]. Hence, the sea turtle habitat is mostly found at the tip of South Africa due to the flow of warm water and absent from the west coast of South Africa due to the flow of cold waters. Nonetheless, a study undertaken by Roberts et al. [45] stated that the clustering and mixing of sea turtles between the Atlantic and Indian Oceans can take place as it is possible for the sea turtle to swim against the current or even swim through the cold water [44,45]. Furthermore, the geographic extent and direction of oceanic migration within a species can vary among populations and among individuals within a population as it is heavily influenced by various factors such as ecological and biogeographical processes, seasonal variation in temperature and current, reproductive needs, and survival patterns across regions [46]. Thus, this provides evidence of migration between the Atlantic and Indian Oceans. This could be another possible explanation from the phylogenetic analysis in this study, where the clustering of sea turtles from the Indian Ocean and Atlantic Ocean are reported. As such, we suggest that a comprehensive study on the migratory routes of the sea turtle population and oceanic current is required to further understand the missing link between geographical connection and ChHV5.

## 3. Materials and Methods

### 3.1. Animals and Ethics Statement

The current study was conducted with the approval of the Universiti Malaysia Sabah (UMS) Institutional Animal Ethics Committee (reference number: UMS/PPPI1.3.2/800-2/1/17Jilid4(06)) and the permission of the Sabah Wildlife Department. All animal experiments and methods were performed in accordance with the relevant local and international regulations and in keeping with the ARRIVE guidelines (https://arriveguidelines.org, accessed on 1 May 2015).

### 3.2. Field Sampling

The field sampling was carried out at Mabul Island (4°14′45″ N 118°37′52″ E) located at the east coast of Sabah, Malaysia, in May and November 2015, and again in November 2016 (Figure 1). Each field trip was conducted for four days, with a total of 13 dives per trip. The study site is a feeding ground for both the Green and Hawksbill turtles. The turtles were caught by hand while SCUBA diving during the day at the seven established dive sites (Figure 1) at depths not exceeding 20 m according to previous established procedure [47]. The turtles were photographed, measured, and tagged on board the research vessel. Two individually numbered Inconel tags from Sabah Wildlife Department, Malaysia were applied to the axial scale of each front flipper [48,49].

**Figure 5:**
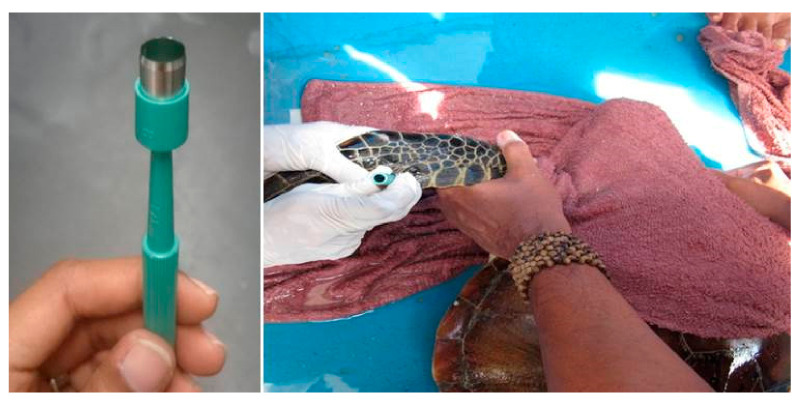
Collection of tissue samples from a juvenile Green turtle from the fore flipper using a 6mm skin biopsy punch.

The turtles were measured using standard procedures [50,51] and all curved carapace length measurements were taken using a flexible tape to the nearest mm. Tissue samples (*n* = 131) from 115 Green turtles and 16 Hawksbills were obtained from the fore flipper using a 6mm skin biopsy punch following the method described by Dutton [52]. Proper disinfectant using betadine was applied at the biopsy site before and after biopsy. The biopsy punch consists of a sharp circular blade (Figure 5). The sample was obtained by pressing down the biopsy punch gently and rotating it on the fore flipper. The circular tissue plug was then removed by forceps and stored at −20 °C (5 days). After transportation to the laboratory, they were kept at −80°C until assayed. The method was recommended to obtain ‘plugs’ of skin and attached sub-epidermal tissue that is adequate for PCR-based genetic analysis. Tissue samples from the tumours were also collected with precaution so that the turtle did not bleed much. As we managed to recapture the same turtle (identified through the tag number) after four months, we noted that the wound was completely healed with no scarring. The turtles were also evaluated physically immediately after capture. All 131 samples were subjected for molecular analysis while three of the turtles with tumours were subjected to histopathological examination. The turtles were then released back into the sea at the captured site after all measurements and tissues were collected.

### 3.3. Histopathological Examinations

Tissue samples from the tumours (*n* = 3) were preserved in a 10% buffered formaldehyde solution prior to fixation and processed for routine histopathology [53] and TEM examination [54]. The tissues were then embedded in paraffin wax. The paraffin-embedded samples were then sectioned to 4 μm and finally stained by haematoxylin and eosin (HE) using standard techniques for histopathological examination. The slide was viewed using light microscopy to confirm the presence of viral particles in the tumour and characterise the tumour morphologically [5,12,16].

### 3.4. Molecular Analysis

DNA was extracted from 25 mg of tissue using a modified cetyltrimethylammonium bromide (CTAB) method [55]. The final purified genomic DNA was eluted in 30 μL of EB buffer. PCR assay was performed with four independent primer sets developed by Alfaro-Núñez et al. [1] and Lu et al. [26] for CFHV detection (Table 2). The primers amplified highly conserved regions of four ChHV5 genes, namely, Capsid protein gene UL18, Glycoprotein H gene UL22, Glycoprotein B gene UL27 [1] and DNA polymerase catalytic subunit pol UL30 [26], the latter have been found to be more sensitive than the other available oligonucleotide primers shown in the previous study [1].

Amplification of the ChHV5 gene regions were carried out set up in a 10 µL reaction volume containing 25–30 ng of genomic DNA, 0.2 U GoTaq DNA polymerase (Promega), 1× GoTaq buffer (Promega), 2.0 mM of MgCl_2_, 10 pmol of each primer, and 0.4 mM of dNTPs in a Thermocycler (PT1000, Bio-Rad Laboratories). The amplification temperature profiles consisted of an initial denaturation at 94 °C for 60 s, followed by 35 cycles of denaturation at 94 °C for 30 s, an optimal annealing temperature for 30 s, extension at 72 °C for 30 s, and a final elongation step at 72 °C for 7 min. The PCR products were visualised on a 1.5% Agarose gel prior to DNA sequencing.

Positive viral amplicons that were identified were further confirmed by Sanger sequencing using the BigDye Terminator Kit v3.1 and analysed on an ABI 3130 DNA sequencer (Applied Biosystems, Inc.). Viral sequences were compared to the Green turtle Herpes virus sequences available in GenBank to verify gene targets according to DNA identity. This was done through multiple sequence alignment by using the ClustalW program implemented in MEGA5.2. Phylogenetic trees were generated using the BEAST v2.50 software with 10,000,000 heated chain generations and a burn-in fraction of 10% [56]. FigTree v.1.44 was used to illustrate the phylogenetic tree [57].

## 4. Conclusions

The results of our study facilitate the understanding of the origin and extent of FP in Mabul Island as the disease is considered to be one of the major threats to *Chelonia mydas*. Turtles infected with FP often do not survive as the tumour obstructs vision and impedes daily activities such as feeding and locomotion. This hampers organ functions and eventually causes death. The 5.22% prevalence in asymptomatic Green turtles in our study implies that despite living in different geographical regions, Green turtles may be exposed to a common pathogen or other environmental factors that aggravate FP. Even though ChHV5 has evolved for millions of years with its turtle host, for the conservation of these endangered sea turtles, ChHV5 needs to be considered as a re-emerging virus, which threatens Green turtles in marine waters surrounding Borneo and worldwide. In particular, the need to gather more information on sea turtle disease risk analysis and disease hazards from various regions is vital. Identification of the cause of FP will be the first step towards developing effective measures for management and control programs.

## Figures and Tables

**Table 1:** (a). Detection of ChHV5 in tissues with and without tumours in the Green and Hawksbill turtles. (b). Green turtle samples identified as positive for the four viral genes verified through DNA sequencing.

(a). Detection of ChHV5 in Tissues with and without Tumours in the Green and Hawksbill Turtles									
Turtle Species		Turtle Captured		Turtles with Tumours (Visual Inspection and Positive Identification Using PCR)			Turtle without Tumours (Positive Identification Using PCR)		
Green		115		3			6		
Hawksbill		16		0			0		
Total		131		9 ^1^					
**(b). Green Turtle ID**									
**Gene**	**FP8 ^2^**	**FP41**	**FP45**	**FP46**	**FP48**	**FP50**	**FP70 ^2^**	**FP71 ^2^**	**FP115**
**Size class**	**Adult Male**	**Juvenile**	**Juvenile**	**Juvenile**	**Juvenile**	**Juvenile**	**Sub-Adult**	**Sub-Adult**	**Juvenile**
Capsid Protein (UL18)	√			√	√		√		
Glycoprotein H (UL22)	√	√	√	√	√	√	√	√	√
Glycoprotein B (UL27)	√	√	√	√		√		√	
DNA Polymerase (UL30)	√	√	√		√				√

^1^ Total number of samples positive for ChHV5. ^2^ Turtle with tumours (visual inspection and positive identification using PCR).

**Table 2:** List of nested primers used to amplify the four ChHV5 gene regions and the expected amplification size (bp).

Region	Primer Name	Primer Sequences 5′–3′	Size (bp)	Reference
Capsid protein gene	UL18 1′	F: CACCACGAGGGGGAAAATGA R: TCAAATCCCCCGTTCACTCG	717	Alfaro-Núñez et al., 2014
	UL18 2′	F: GTGGAACCCCGCCGGGTAAT R: TGATCCGGGCCGAGTAGCGG	140	
Glyco-protein H gene	UL22 1′	F: ACGGCGTTGGCTAGTGAATC R: GCAGTTCGGTACACACCTCT	386	Alfaro-Núñez et al., 2014
	UL22 2′	F: AACGCCCTTTCCTCCGACCCATATT R: GCTGGGGGAGCATCGTGCAAA	179	
Glyco-protein B gene	UL27 1′	F: TAACAAGAAAGAACCGCGCG R: ATTTTCCCGGTCAGTGCCAA	352	Alfaro-Núñez et al., 2014
	UL27 2′	F: CTAGATACATACTGGCCRTGCTCGTC R: GCCAGCGACCATCCGGAG	143	
DNA Polymerase Subunit Pol gene	UL30 1′	F: AGCATCATCCAGGCCCACAATCT R: CGGCCAGTTCCGGCGCGTCGACCA	445	Lu et al., 2000
	UL30 2′	F: AGCATGTCGCGCCCTACGGTGGTGAC R: CTGCTGACCGACTGGCTGGC	206	

## Data Availability

All genomic data are available at GenBank database; accession numbers for the sequenced genomes are listed in the main text. The datasets generated during and/or analysed during the current study are available from the corresponding author.
